# Human Saliva Collection Devices for Proteomics: An Update

**DOI:** 10.3390/ijms17060846

**Published:** 2016-06-06

**Authors:** Zohaib Khurshid, Sana Zohaib, Shariq Najeeb, Muhammad Sohail Zafar, Paul D. Slowey, Khalid Almas

**Affiliations:** 1 College of Dentistry, King Faisal University, Al-Hofuf, Al-Ahsa 31982, Saudi Arabia; drzohaibkhurshid@gmail.com; 2 Department of Biomedical Engineering, School of Engineering, King Faisal University, Al-Hofuf, Al-Ahsa 31982, Saudi Arabia; szohaib@kfu.edu.sa; 3 Restorative Dental Sciences, Al-Farabi Colleges, Riyadh 361724, Saudi Arabia; shariqnajeeb@gmail.com; 4 Department of Restorative Dentistry, College of Dentistry, Taibah University, Al Madinah, Al Munawwarah 41311, Saudi Arabia; 5 Oasis Diagnostics^®^ Corporation, 15720 NE 31st Avenue, Vancouver, WA 98686, USA; pds@4saliva.com; 6 Division of Periodontology, College of Dentistry, University of Dammam, Dammam 31441, Saudi Arabia; Kalmas@uod.edu.sa

**Keywords:** omics, salivaomics, proteomics, salivary biomarkers, saliva collection devices

## Abstract

There has been a rapid growth in the interest and adaptation of saliva as a diagnostic specimen over the last decade, and in the last few years in particular, there have been major developments involving the application of saliva as a clinically relevant specimen. Saliva provides a “window” into the oral and systemic health of an individual, and like other bodily fluids, saliva can be analyzed and studied to diagnose diseases. With the advent of new, more sensitive technologies to detect smaller concentrations of analytes in saliva relative to blood levels, there have been a number of critical developments in the field that we will describe. In particular, recent advances in standardized saliva collection devices that were not available three to four years ago, have made it easy for safe, simple, and non-invasive collection of samples to be carried out from patients. With the availability of these new technologies, we believe that in the next decade salivary proteomics will make it possible to predict and diagnose oral as well as systemic diseases, cancer, and infectious diseases, among others. The aim of this article is to review recent developments and advances in the area of saliva specimen collection devices and applications that will advance the field of proteomics.

## 1. Introduction

The presence of nucleic acids (DNA, RNA), proteins, peptides, and other components in human bodily fluids makes them ideal media for disease diagnosis. Indeed, in the clinical environment bodily fluids such as blood, urine, cerebrospinal fluid (CSF), peritoneal fluid, vomit, sweat, tears, breast milk, semen, vaginal fluids, and drainage fluids are used in a significant proportion of tests available in the *in vitro* diagnostic marketplace [1]. The differing compositions in each bodily fluid requires that “fluid-specific” devices need to be applied for specimen collection and analysis [2].

“Omics” is a broad collection of technologies used to explore the biochemistry, role, relationship, and the action of various types of molecules that make up the cells of organisms and the term includes genomics (the study of genes), transcriptomics (the study of mRNA within cells or organisms), metabolomics (the study of global metabolite profiles in a system under a given set of conditions), and proteomics (the study of proteins) [3]. The basic aim of these technologies is to understand complex physiological processes in normal healthy individuals and diseased patients. The term “Salivaomics” was first introduced in 2008, on the basis of the discovery of specific biomolecules appearing in the saliva. These included DNA, mRNA, microRNA, proteins, metabolites, and oral microbiota extractions [4]. This pioneering work was based on the extraction of DNA from preoperative/postoperative saliva and soft tissue samples taken from patients with oral squamous cell carcinoma in comparison to salivary DNA extracts taken from healthy subjects, used as a control group. This particular study highlighted marked differences in DNA-methylation patterns between the samples [5]. Li *et al.* used microarray technology to identify RNA profiling in saliva and opened up the window for the compilation of a reference database for diagnostic applications using the salivary transcriptome [6].

A number of salivary transcriptomic biomarkers have been identified that appear to be up- or down-regulated in human malignancies (ovarian cancer, lung cancer, and oral squamous cell carcinoma) and these are related to various biochemical processes, which includes growth regulation by estrogen in breast cancer 1, brain acid-soluble protein 2, β-2-microglobulin, immediate early response 3, IL1B, IL-1β, IL-8, S100 calcium binding protein-P, spermidine/spermine *N*^1^ acetyltransferase-1, epidermal growth factor receptor, cyclin 1, fibroblast growth factor 19, and fibroblast growth factor substrate 2 [7,8,9].

Wang *et al.* used ultra-performance liquid chromatography (UPLC) mass spectrometry in a hydrophilic interaction chromatography (HILIC) mode—and identified choline, betaine, pipecolinic acid, l-carnitine, l-leucine, and l-phenylalanine in saliva using a metabolomics approach, and from this study the authors were able to report a novel metabolite biomarker for the early diagnosis of squamous cell carcinoma [10,11]. Overall, with the aid of proteomic approaches researchers have been able to identify over 1000 salivary protein biomarkers including secretory immunoglobulin A, lactoperoxidase, statherin, proline-rich glycoprotein, truncated cystatin S, cystatins, lysozyme, and histatin-5, among others [12,13,14,15,16,17].

With new advancements in the proteomic sciences, including mass spectrometric techniques that detect low levels of analytes in sample matrices, it has become significantly easier to analyze human salivary proteins and peptides [18]. Human saliva is an ultra-filtrate of blood and represents the physiology of the whole human body through its composition (DNA, RNA, proteins, metabolites, and microbes) [19]. Human saliva also aids in a number of other bodily functions including food tasting, swallowing, digestion, oral protection, oral defense, and maintenance of tissues, but over the last 10 years, the increased application of saliva as a medium for disease diagnosis has become much more evident [20].

The aim of this review is to highlight the growing importance of human saliva as a diagnostic medium, the availability of new and standardized tools for saliva specimen, and general protocols for saliva sampling, storage, and processing, which have changed significantly since the last reviews on this subject. For a more detailed overview of salivary diagnostics, potential applications, and tools, the reader is referred to several excellent reviews on this subject [21,22,23,24,25].

## 2. Human Salivary Proteomics

Human whole mouth fluid (WMF) is a broad term which includes saliva, gingival crevicular fluids (GCF), oral mucosal transudate, mucus from the nasal cavity and the pharynx, as well as products resulting from bacterial metabolism, food debris, and desquamated epithelial cells [26]. This critical bio-fluid not only provides lubrication, protection, oral defense, and aids in speech, but it has also been recognized as one of the most important bodily fluids for the diagnosis of diseases, both oral and systemic. It is important at this point to highlight the basic differences between several terms, particularly whole mouth fluid (WMF), saliva, and gingival crevicular fluid (GCF). Whole mouth fluid (WMF) is a term used interchangeably with “saliva”, (including whole mouth saliva [WMS] used in this manuscript) and is used in the forensic sciences and toxicology [27].

Saliva is secreted by three pairs of major salivary glands (the parotid, submandibular, and sublingual glands) and also receives contributions from 300 to 400 minor salivary glands present in the oral cavity [28]. Biochemical analysis of human saliva reveals that it is composed of inorganic constituents (sodium, potassium, calcium, magnesium, chloride, and phosphate), and organic constituents (proteins and non-proteic components) [29]. The protein components of human saliva include amylase, mucins, lysozyme, IgA, lactoferrin, proline-rich proteins, histatins, cathelicidins, defensins, glycoproteins, lipoproteins, statherin, and matrix metalloproteases [14,30]. The non-proteic components include bilirubin, creatinine, glucose, and uric acids [31]. As mentioned earlier, over the last two decades, human saliva has been increasingly used as a biofluid for the diagnosis and prognosis of oral diseases, and systemic diseases including auto-immune diseases, human immunodeficiency virus (HIV), cancer, and others [32]. In the recent outbreak of the Zika virus, researchers were able to readily detect Zika virus (ZIKV) RNA in the saliva of patients in the acute stage of the disease [33]. This followed on from previous studies in French Polynesia that reported the presence of ZIKV in the saliva of a mother and her infant [34].

There are many properties of human saliva that attract clinicians or researchers to adopt the use of saliva specimens and reinforce the use of this non-invasive fluid in diagnostic algorithms. Some of these are highlighted below:

- Non-invasive
- Simple collection protocols
- Non-infectious sample
- Easily disposal
- Easily transportable
- Cost effective
- Not subject to cultural and religious “taboos”
- Safe and effective
- Higher patient compliance.

**Figure 1:**
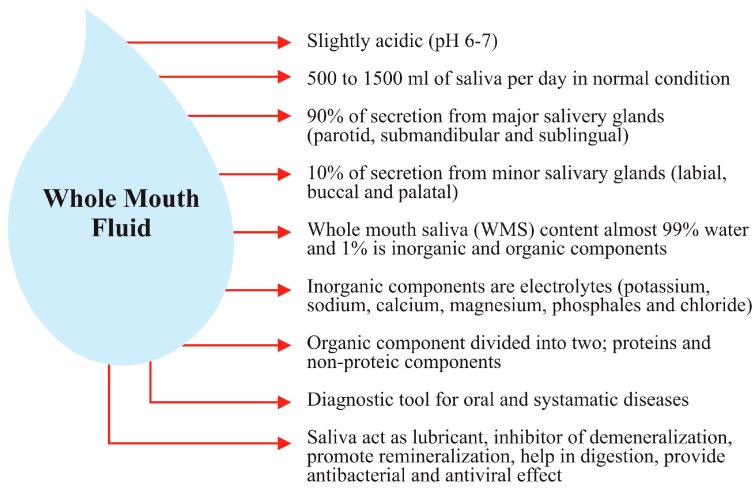
Illustration of whole mouth saliva representing the properties of saliva.

Some of the important properties of saliva are further illustrated below in Figure 1.

Salivaomics is the study of salivary “omics” methodologies including the genome, the epigenome, the transcriptome, the proteome, the microbiome, and the metabolome [4]. The capability to collect a sample in a non-invasive, safe, and cost effective fashion, with the benefits of higher patient comfort and compliance makes the adoption of saliva for each of these “omics” techniques an attractive proposition for all parties concerned (patients, researchers, and clinicians) [35].

Saliva contains greater than 2000 proteins and peptides and these are involved in a multitude of different biological functions in the oral cavity. It is these characterized proteins and peptides that can be analyzed to monitor or identify various pathologies in humans. In the last 20 years the ability to detect and measure proteins both qualitatively and quantitatively using novel proteomics technologies has brought about a “quiet revolution” in the detection of diseases using various protein biomarkers in saliva [36,37].

As part of the overarching goals for proteomics technologies, it is important to investigate the diverse and enabling properties of proteins. The means of achieving this goal has been achieved in part using sensitive and highly accurate analysis by high throughput techniques such as mass spectrometry (MS), gas chromatography/mass spectrometry (GC-MS), high-pressure liquid chromatography (HPLC), and two-dimensional liquid chromatography (2D-LC). A very recent addition to this list surface-enhanced laser desorption/ionization (SELDI) MS based ProteinChip technology has also moved this field forward significantly [38,39,40,41].

As part of our database searching to enable writing this manuscript, we searched the following biomedical databases: PubMed, Google Scholar, and Scopus. We searched under the keywords “salivaomics”, “proteomics”, and “salivary biomarkers” to retrieve recent publications in the area from 2005 up to the present day. As a result of the search we further considered only publications pertaining to human saliva proteomics and saliva sample collection devices.

## 3. Human Saliva Collection Devices

The very first instance of a method for saliva collection from a patient was in the early 19th century (1934) by Wainwright for the analysis of salivary calcium (Ca^2+^). In Wainright’s method, the patient’s head was tipped forward with the mouth pointing vertically downwards and saliva was allowed to drip from the mouth into a filter funnel [42].

Normal healthy adults produce around 0.5 to 1.5 liters of saliva per day (or approximately 0.5 mL/min) but in various systemic diseases, and in pathological and physiological conditions, there may be a considerable (negative) impact on the salivary flow rate [43]. There are three main types of human saliva (and in addition several sub-component fractions) and the method of collection of each type varies accordingly, as described previously (See Table 1).

In the last 30 years salivary diagnostics have increasingly gained in attention and the discipline has now become widely employed in the biomedical and clinical sciences. One of the reasons for this is the capability to identify various proteins in saliva, which can be accomplished via proteomic technologies. Two companies from the United States, namely Epitope, Inc. (Oregon, now OraSure Technologies, Bethlehem, PA, USA) and Saliva Diagnostic Systems, Inc. (Vancouver, WA, USA), were two of the early pioneers in the area; each developed commercially viable saliva collection devices in the 1990s, and each of these has been used in proteomic analysis and other areas of research and clinical practice. The OraSure Device from Epitope/OraSure was the first saliva collection device to be linked to a clinical test for the human immunodeficiency virus (HIV) and the company was successful in gaining Food and Drug Administration (FDA) approval for the device in conjunction with a laboratory enzyme-linked immunosorbent assay (ELISA) test for the HIV virus. Saliva Diagnostic Systems’ Saliva-Sampler^®^ device was cleared for marketing by the FDA as a general purpose saliva collection device without being tied to a specific clinical application. These early devices spurred new developments in saliva collection device technology resulting in devices that produce “cleaner” specimens, thereby allowing clinicians to analyze saliva more easily than before. Other devices that have been used historically include the Salivette device from Sarstedt, and the Salimetrics Oral Swab (SOS).

Currently, new innovations in saliva collection are used as integral parts of tests for HIV testing, rapid drugs of abuse tests, medical testing for life insurance, and for isolating DNA/RNA and proteins for genomic, transcriptomic, and proteomic analysis, respectively.

**Figure 2:**
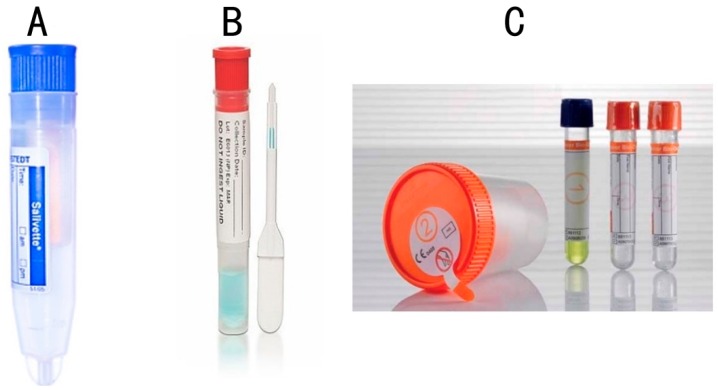
A selection of different saliva collection devices used in medical and dental research. (**A**) Salivette^®^ (Sarstedt); (**B**) Quantisal ^®^ (Immunalysis); (**C**) SCS^®^ (Greiner-BioOne).

By way of examples of some studies, Gröschl *et al.* [51] evaluated steroids, peptides, and therapeutic drugs from saliva samples using the Salivette^®^ device (Sarstedt, Nümbrecht, Germany, Figure 2A), Quantisal^®^ (Immunalysis, Pomona, CA, USA, Figure 2B), and SCS^®^ (Greiner-BioOne, Kremsmünster, Austria, Figure 2C) devices and concluded that immediate processing of the sample is important to avoid errors in the study due to sample instability issues. The authors of this study also commented on the adsorption behavior of saliva on various devices, which could negatively impact sample integrity [51].

A separate group of researchers used the Salimetrics^®^ Oral Swab (SOS), Salivette^®^ (cotton and synthetic devices, Sarstedt), and Greiner Bio-One Saliva Collection Devices (GBO SCS^®^) in a study analyzing salivary C-reactive protein (CRP), IgE, and myoglobin levels in healthy participants. Depending upon the collection method used, significant differences in analyte levels were observed [52]. These findings suggest a requirement for suitable devices that provide accurate analyte levels that correlate with levels observed in “gold standard” technologies, particularly passive drool.

Speicher *et al.*, performed an experiment on oral fluids to detect DNA viruses using the DNA Genotek OMNIgene™ DISCOVER kit, and concluded that the kit is suitable for collection, long term storage, and genomic assays [53]. These are just a few of the significant number of studies that have been performed on saliva specimens [53,54,55,56,57].

**Figure 3:**
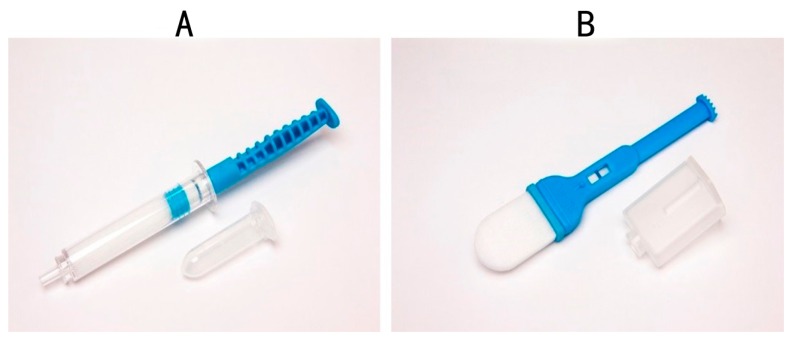
Saliva Collection Device manufactured by Oasis Diagnostics^®^ Corporation; (**A**) Super•SAL™ and (**B**) Versi•SAL^®^.

Newer devices on the market “mimic” whole saliva collection using passive drool. These devices, which includes the Super•SAL™ (Figure 3A) and Versi•SAL^®^ (Figure 3B) technologies (Oasis Diagnostics^®^ Corporation, Vancouver, WA, USA), use inert absorbent materials as the collection media that effectively release analytes from the pad, in contrast to cotton based collection methods that are reported to cause interference with certain biomarkers in saliva [51,54]. In addition, these newer devices provide a standardized specimen, resulting in more consistent sample uniformity. The sample obtained from either of these devices is a neat sample, meaning that there are no buffers required to dilute the specimen. In the case of earlier devices (OraSure, Saliva-Sampler^®^), a buffer is included and, in some cases, there is a requirement to centrifuge the sample before processing. In the case of the neat sample collected by the Super•SAL™ and Versi•SAL^®^ Devices, the sample can be immediately tested without any additional manipulation steps. Super•SAL™ and Versi•SAL^®^ also incorporate a Sample Volume Adequacy Indicator (SVAI) that provides a visual indication of when sample collection is complete. In the case of the Saliva-Sampler^®^ device, this also has an SVAI, but in the case of devices such as the OraSure^®^, Salivette^®^, and SOS Devices, there is no means of determining sample adequacy.

Most devices mentioned in this manuscript collect a minimum of 0.5 mL of saliva and the same is true for the newer devices. The typical yields of whole saliva from Super•SAL™ and Versi•SAL^®^ are 1.0 and 1.2 mL, respectively. The quality of the saliva obtained is much higher than passive drool, since in the case of these devices, the absorbent pad material used to collect the saliva specimen also acts as a filter to remove large mucinous material that can cause downstream assay interferences.

These devices (Figure 3A,B) have been used successfully to collect hormones [58], proteins, and biomarkers potentially useful in the diagnosis of Parkinson’s disease [54] as well as infectious agents including the Ebola virus [59] and Lassa fever. In the current situation, where there are multiple global epidemics caused by different viruses (e.g., Ebola, Dengue fever, and Zika virus); these devices for accurate and standardized saliva collection can play a role in the early diagnosis of viral diseases that can save many lives. On 1 February 2016, the World Health Organizations (WHO) declared a global emergency for Zika virus in an attempt to limit the spread of the lethal virus. Later in 2016, there will be a mass gathering at the Olympic Games in Brazil Olympics, a country where the Zika virus is a significant issue. In addition, venues such as Ummrah, and the Hajj in Saudi Arabia, where multiple gatherings converge, may be ideal venues to implement screening for the Zika virus through simple and effective saliva sampling and subsequent screening of visitors for the fatal virus. It is important to design new strategies to reduce the spread of Zika virus (ZKV) and other virulent diseases in order to keep our population safe and saliva is the ideal specimen to be used in these situations [60]. The critical thing is that the latest generation of devices provide a standardized sample of saliva, representative of whole mouth saliva.

## 4. Conclusions

Over the last few years, the importance of saliva has grown rapidly for both research and clinical applications. This is due to a number of advantages that saliva offers, particularly non-invasive sampling, higher patient compliance, easy, simple collection, and cost, among others. Salivary diagnostics are already available for nucleic acid testing, drugs of abuse monitoring, and general wellness testing among others, and assays are very close to market for targets in the Sjögren’s syndrome area looking at various proteins. In addition, clinical tests using mass spectrometry for steroid hormones (e.g., cortisol and testosterone) are already performed on saliva specimens in significant volumes in the large reference laboratories in the United States.

We believe that in the near future, salivary proteomics will be used synergistically for protein analysis as with other bodily fluids such as nipple aspirate, urine, blood, cerebrospinal fluid (CSF), and tears. Recent advances, particularly in the standardization of collection of specimens using saliva collection devices, have made it easy for safe, simple, and non-invasive collection of samples from patients. We believe that in the next decade salivary proteomics will become an even more valuable tool for the prediction and diagnosis of oral, as well as systemic diseases, due to the availability of highly effective saliva collection devices, adding further value to the role that salivary diagnostics plays in delivering patient-centric healthcare.

## Figures and Tables

**Table 1:** Description of Human Saliva Collection Methods.

Type of Whole Mouth Fluid	Method of Collection and Type of Collection Device	References
Whole Saliva (WS)	Patients should refrain from eating, drinking, and oral hygiene procedures for at least 1 h before saliva collection. (Optimum collection time is 8–10 a.m.). Before collection perform a 1 min oral rinse with distilled water and then after 5 min collect ~5 mL of saliva. Collected sample must be processed in the laboratory within 1 h.	[44]
Unstimulated Whole Saliva (USWS)	Passive drooling: In this method restrict oral movement and drain saliva from the lower lip into a plastic vial.	[45]
	Spitting method: Instruct subject to spit into a collection vial. In this method 14 times more bacterial contamination is introduced into the sample.	
Stimulated Whole Saliva (SWS)	For the stimulation of glands, chewing different things like natural gum, a piece of paraffin wax, citric acids, and powdered drink crystals have been used.	[46,47]
Parotid Gland	Method introduced by Carlson and Crittenden (1910). In this method a double chambered metallic cup with two outlet tubes is used. One end holds the cup in place using vacuum suction. The second half acts as a collection vehicle for saliva. Specimen collection can be enhanced by smearing citric acid (10%; 1 mL) on the dorsum of tongue every 30 s. Discard the first 1.5 mL of saliva prior to sample collection.	[48]
Submandibular/Sublingual Gland	Truelove, Bixler, and Merrit (1967) used a “V”-shaped collector. This method is similar to that for parotid gland collection, but in this case the initial 2 mL is discarded.	[49]
Minor Glands	Kutscher *et al.* (1967) used capillary tubes for collecting saliva from minor glands located at the everted surface of the lower lips.	[50]
